# Programmed Sequential Release Behavior and Bioavailability of Curcumin and Fucoxanthin Co‐Encapsulated in Solid‐In‐Oil‐In‐Water Multilayer Emulsions

**DOI:** 10.1002/fsn3.71463

**Published:** 2026-01-19

**Authors:** Luhui Wang, Mingqing Wang, Ling Lv, Changhu Xue

**Affiliations:** ^1^ Shandong Peanut Research Institute Shandong Academy of Agricultural Sciences Qingdao China; ^2^ College of Food Science and Engineering Ocean University of China Qingdao China

**Keywords:** bioavailability, co‐delivery system, in vitro digestion model, programmed sequential release, solid‐in‐oil‐in‐water multilayer emulsion

## Abstract

Food‐grade co‐delivery systems have garnered significant attention for their ability to deliver two or more bioactive components simultaneously. Co‐delivery systems possessing programmed sequential release properties allow sequential delivery of bioactive components to different sites in the gastrointestinal tract (GIT) to enhance their bioavailability. This study constructed solid‐in‐oil‐in‐water multilayer emulsions (S/O/W‐E) using carboxymethyl konjac glucomannan‐coated gliadin nanoparticles as the solid phase, coconut oil as the oil phase, and carboxymethyl starch/propylene glycol alginate complexes as the aqueous phase, which realized the co‐encapsulation of curcumin (Cur) and fucoxanthin (FUC) and their programmed sequential release in the GIT. The programmed sequential release behavior of S/O/W‐E was further evaluated by in vitro digestion models. It was demonstrated that both Cur and FUC released less than 16.5% in simulated gastric fluid, following a Fickian diffusion. The Cur located in the oil phase was released in large quantities (67.3%) in simulated intestinal fluid, predominantly through erosion. Owing to the action of β‐mannanase, 60.3% of FUC located in the solid phase was released into simulated colonic fluid and dominated by the erosive mechanism. In addition, in vivo bioavailability evaluation and fluorescence imaging experiments confirmed that S/O/W‐E enhanced Cur bioavailability by 6.4‐fold through delivering it to the small intestine and inhibited FUC release in the upper GIT by delivering substantial amounts of FUC to the colon. This study is beneficial for effectively expanding the application of S/O/W‐E in co‐delivery systems.

## Graphical abstract

Solid‐in‐oil‐in‐water multilayer emulsions with programmed sequential release performance enhanced the bioavailability of bioactive components in the oil phase and increased the colon distribution of bioactive components in the solid phase.

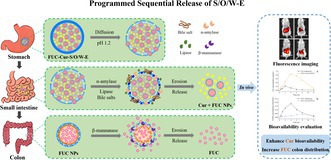

## 1 Introduction

As consumers become more health‐conscious, the preparation of functional foods containing food‐grade bioactive components has become a hot research topic. Along with the deepening research on the synergistic effects among bioactive components, the co‐encapsulation of multiple bioactive components has also become a mainstream direction for exploring functional foods (Dima and Dima 2020; Leena et al. 2022). Designing and exploring functional foods with diverse bioactive components and health benefits is not only of practical significance but also poses new requirements and challenges for constructing food‐grade co‐delivery systems (Liu et al. 2022).

Currently, a significant amount of research is focused on the construction of gastrointestinal tract (GIT) single‐targeted co‐delivery systems, which are capable of delivering encapsulated bioactive components to a single target site within the GIT while maintaining the desired concentration and achieving controlled release (Tie and Tan 2022; Zheng et al. 2017). Nevertheless, various food‐grade bioactive components play various physiological functions in different sites of the GIT. For instance, curcumin (Cur), a natural polyphenolic compound extracted from turmeric, exerts beneficial effects on small intestinal health by protecting and repairing the intestinal barrier (Rajasekaran 2011). Fucoxanthin (FUC) is a natural carotenoid derived from brown algae, with extensive research revealing its significant potential for colon health, particularly in anti‐inflammatory effects, colon cancer prevention, and gut barrier protection (Liang et al. 2022; Terasaki et al. 2019). Selecting carrier materials with GIT‐specific degradation properties to construct co‐delivery systems for encapsulating and delivering multiple bioactive components enables them to be absorbed and utilized at various corresponding sites in the GIT, thereby maximizing their beneficial effects. Based on this, the GIT co‐delivery system possessing programmed sequential release performance was constructed (Wang et al. 2023, 2024), which can sequentially deliver the co‐encapsulated bioactive components to different sites of the GIT to exert the synergistic efficacy among the bioactive components and their respective bioavailability in vivo.

We have constructed solid‐in‐oil‐in‐water multilayer emulsions (S/O/W‐E) by utilizing structural design principles using carboxymethyl konjac glucomannan‐coated gliadin nanoparticles (Gli‐CMK NPs) with colon‐targeted delivery performance (Wang et al. 2022a) as the solid phase (S phase), coconut oil as the oil phase (O phase), and carboxymethyl starch (CMS)/propylene glycol alginate (PGA) complexes with small intestinal environmental responsive properties (Wang et al. 2022b) as the aqueous phase (W phase). Previously, Cur and FUC, which exert different beneficial effects on the small intestine and colon, were used as model bioactives of S/O/W‐E, and the GIT programmed sequential release properties were preliminarily confirmed (Wang et al. 2024). However, there have been no reports available on the programmed sequential release behavior of S/O/W‐E. In fact, changes in S/O/W‐E during digestion, along with the release kinetics and mechanisms of the encapsulated bioactive components and their bioavailability in vivo, are crucial for the application of S/O/W‐E in co‐delivery systems.

An in vitro digestion model is an invaluable means in elucidating potential release mechanisms of delivery systems (Malekjani and Jafari 2022), and a thorough understanding of these mechanisms can aid in designing and constructing suitable delivery systems to regulate the delivery behavior of bioactive components. To this end, Cur and FUC co‐encapsulated S/O/W‐E (FUC‐Cur‐S/O/W‐E) were prepared on the basis of our previous studies, and droplet characteristics and morphological changes of S/O/W‐E were measured before and after simulated gastrointestinal digestion. The release behavior of S/O/W‐E into different formulations of simulated gastrointestinal fluids was explored. And then, the release kinetics and mechanisms of S/O/W‐E in simulated gastric fluid (SGF), simulated intestinal fluid (SIF), and simulated colonic fluid (SCF) were evaluated with Zero‐order, First‐order, Higuchi, and Korsmeyer‐Peppas kinetic models. Additionally, DIR was used as a fluorescence tracer instead of bioactive components to observe the intestinal distribution of S/O/W‐E in mice via an in vivo imaging system. Finally, bioavailability of the encapsulated bioactive components was assessed by determining the pharmacokinetic parameters in mice after gavage of S/O/W‐E. This work elucidates the programmed sequential release behavior of bioactives co‐encapsulated in S/O/W‐E under simulated gastrointestinal conditions and their in vivo bioavailability, which facilitates the application of S/O/W‐E for the co‐delivery of multiple bioactives.

## 2 Materials and Methods

### 2.1 Materials

Gliadin (Gli, protein content 91.2%) and coconut oil were provided by Shanghai Macklin Biochemical Co. Ltd. (Shanghai, China). Carboxymethyl konjac glucomannan (CMK, DS 0.55, Mw 4.8 × 10^5^ Da) was obtained as published previously (Wang et al. 2019). Carboxymethyl starch (CMS, DS 0.35) was provided by Aladdin‐reagent Co. Ltd. (Shanghai, China). Propylene glycol alginate (PGA, purity 98%, DE 75%) was supplied by Shanghai Yuanye Biotechnology Co. Ltd. (Shanghai, China). Fucoxanthin (FUC, purity ≥ 80%) was provided by Shandong Jiejing Group Co. Ltd. (Rizhao, China). Curcumin (Cur, purity ≥ 95%) was supplied by Shanghai Ryon Biological Technology Co. Ltd. (Shanghai, China). Fluorescence dye (DIR) was provided by Yeasen Biotechnology Co. Ltd. (Shanghai, China).

### 2.2 Fabrication of FUC‐Cur‐S/O/W‐E

**FIGURE 1:**
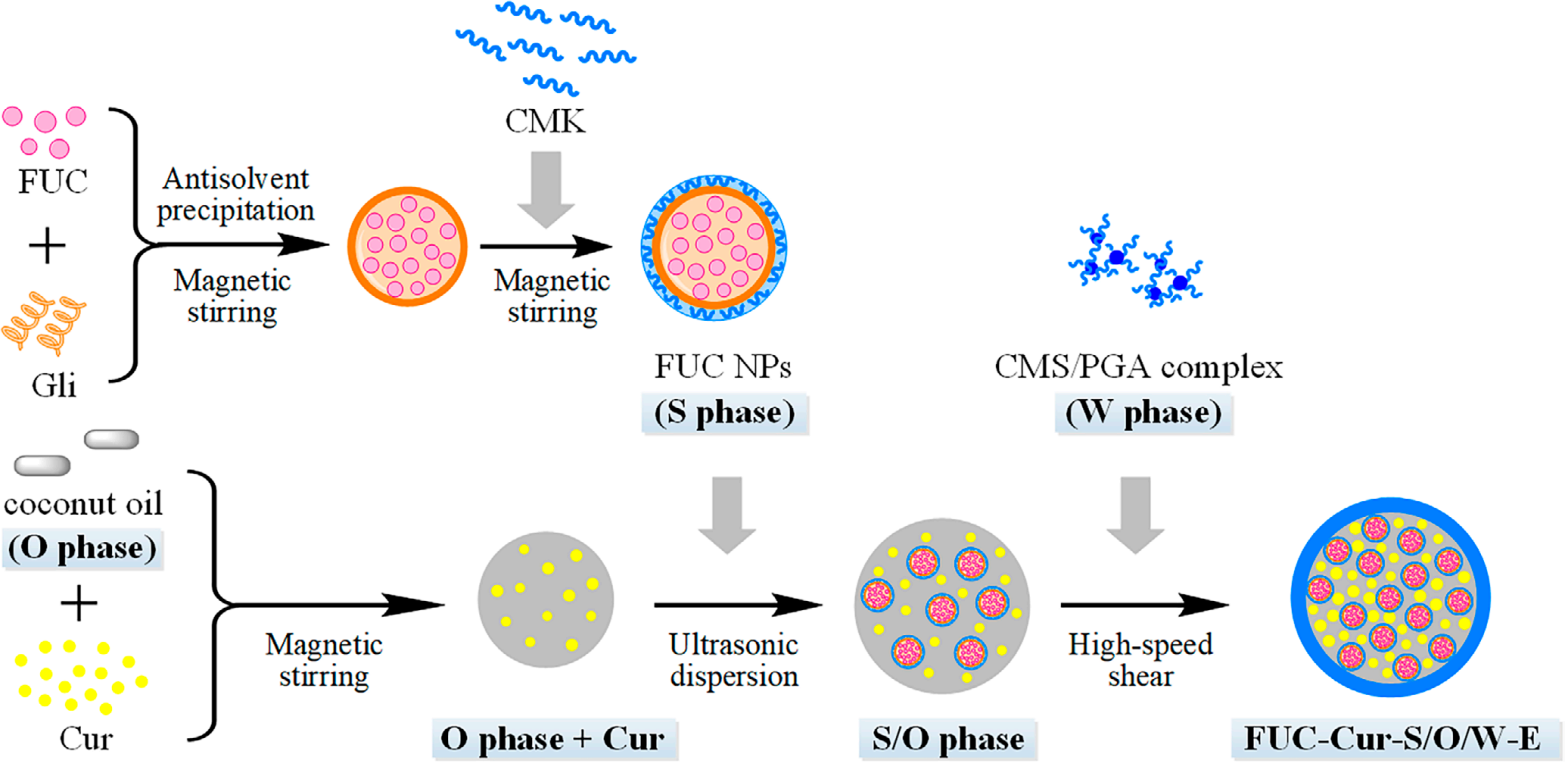
Preparation flow chart of FUC‐Cur‐S/O/W‐E.

The preparation protocol of S/O/W‐E referred to our previous method (Wang et al. 2024). Initially, Gli‐CMK NPs encapsulating FUC (hereafter referred to as FUC NPs) were prepared as the S phase by an antisolvent precipitation method (Wang et al. 2022a). A 70% ethanol solution mixed with 20 mg/mL Gli and 2 mg/mL FUC was added drop‐by‐drop at a volume ratio of 1:9 to pure water at pH 3.0 with a magnetic stirrer, and then an equivalent volume of CMK solution (pH 3.0) was added drop‐by‐drop (Gli/CMK mass ratio 1:1) to fabricate FUC NPs. Ethanol was evaporated by rotary evaporation at 40°C, and the lyophilized FUC NPs were treated as the S phase. Next, PGA was added to the CMS dispersion, and CMS/PGA complexes were obtained as the W phase at a CMS/PGA mass ratio of 4:1 and stirred continuously overnight at room temperature (Wang et al. 2022b). Afterwards, 2.5 mg/mL Cur was dissolved in coconut oil (O phase) with continuous magnetic stirring, followed by adding 10 mg/mL lyophilized FUC NPs for ultrasonic dispersion and refrigerated at 4°C to fabricate the S/O phase. At last, the S/O phase was added to the W phase at a volume ratio of 2:8 and high‐speed sheared (13,000 rpm, 3 min) to form FUC and Cur co‐encapsulated S/O/W‐E (FUC‐Cur‐S/O/W‐E). The preparation flow chart of FUC‐Cur‐S/O/W‐E is depicted in Figure 1.

### 2.3 Characterization of FUC‐Cur‐S/O/W‐E

The droplet size and size distribution of FUC‐Cur‐S/O/W‐E was detected using a laser particle size analyzer (Mastersizer 3000, Malvern, UK). The microstructure of FUC‐Cur‐S/O/W‐E was characterized using a microscope (Ni‐E, Olympus, Japan).

### 2.4 Release Behavior of S/O/W‐E in Simulated Gastrointestinal Conditions

In vitro simulated digestions of FUC‐Cur‐S/O/W‐E at SGF, SIF and SCF were performed according to previous methods (Wang et al. 2024) with some modifications. SGF (pH 1.2) was prepared by dissolving 9600 U/L pepsin in hydrochloric acid solution. SIF (pH 6.8) was formulated by adding 25,000 U/L trypsin, 1.6 mg/mL lipase, 5 mg/mL bile salts and 10 U/mL α‐amylase to phosphate buffer solution, in which the volume ratio of amyloglucosidase to SIF was 1:120. SCF (pH 7.4) was formed by adding 6000 U/L β‐mannanase to phosphate buffer solution.

FUC‐Cur‐S/O/W‐E were mixed in equal volume with SGF, then the pH was adjusted to pH 1.2 and shaken at 37°C to simulate gastric digestion. After 2 h of digestion in SGF, the digestive solution was adjusted to pH 6.8, and then an equal volume of SIF was added to simulate intestinal digestion at 37°C with shaking. After 3 h of digestion in SIF, the digestive solution was adjusted to pH 7.4, and then an equal volume of SCF was added to simulate colonic digestion. The volume ratios of S/O/W‐E: SGF: SIF: SCF were 1:1:2:4. At each designated time, a portion of the digestive solution was removed, and the FUC‐Cur‐S/O/W‐E in the simulated digestion process was characterized.

Bioactives (FUC and Cur) released from FUC‐Cur‐S/O/W‐E were extracted by methanol after centrifuging at 10,000 g for 30 min, and the content of bioactives was assayed by high‐performance liquid chromatography (HPLC). For FUC, the mobile phase consisted of water (A) and methanol (B) in an isocratic elution program (10% A: 90% B) with a flow rate of 1.0 mL/min at 30°C (Hashimoto et al. 2009), and eluents were assessed at a wavelength of 450 nm. For Cur, the gradient elution was carried out with the mobile phase consisting of 4% (v/v) acetic acid (A) and acetonitrile (B) (0.01–10 min, 30%–100% (B), 10.01–15 min, 100%–30% (B)) (Wichitnithad et al. 2009). The column temperature was set at 25°C, the wavelength was 425 nm, and the flow rate was 1.0 mL/min. All chromatographic separations were achieved using a C18 analytical column (150 × 4.6 mm) with 5 μm particle size. The bioactive release rate was expressed as the percentage of released bioactives to the total bioactives encapsulated in FUC‐Cur‐S/O/W‐E.

### 2.5 In Vitro Release Kinetics of S/O/W‐E

Release kinetics of S/O/W‐E were exploited by fitting the release data in simulated gastrointestinal fluids with zero‐order, first‐order, Higuchi and Korsmeyer‐Peppas release kinetic models (Ritger and Peppas 1987; Tallarida 2001). These selective models are frequently employed to describe the bioactives release from the delivery system when the release mechanism is unfamiliar or involves multiple release scenarios. The model with the highest correlation coefficient (*R* ^2^) was judged to be the optimal kinetic model for Cur and FUC release in S/O/W‐E.

In the zero‐order model, the bioactives release rate is independent of concentration, whereas the first‐order model is concentration‐dependent. Higuchi model is classically used to assess whether the release is primarily governed by Fickian diffusion through a matrix. Korsmeyer‐Peppas model helps to identify specific release mechanisms (e.g., Fickian diffusion, Case‐II transport, or anomalous transport) from colloidal delivery systems. The equations are as follows:

(1) Zero−order model:Mt/M∞=kt

(2) $$\text{First}-\text{order model}:\ln\left(1-\mathrm{Mt}/M\infty \right)=-\mathrm{kt}$$

(3) $$\text{Higuchi model}:\mathrm{Mt}/M\infty ={\mathrm{kt}}^{1/2}$$

(4) $$\text{Korsmeyer}-\text{Peppas model}:\mathrm{Mt}/M\infty ={\mathrm{kt}}^{n}$$

in which *Mt*/*M*∞ denotes the cumulative release amount of bioactives at *t*, *k* represents the rate constant, and *n* refers to the diffusional exponent.

### 2.6 In Vivo Fluorescence Imaging in Mice

S/O/W‐E were prepared by substituting the bioactive components with DIR, and those substituted only for FUC or Cur were named DIR‐S/O/W‐E or S/O/W‐E‐DIR, where the DIR concentration was 0.1 mg/mL in both cases. After adaptive feeding for 1 week, C57BL/6 mice (Male, 6 weeks old, 20 ± 2 g) were shaved of abdominal fur and divided into three groups (three mice per group), and each group was gavaged with 0.1 mL of free DIR, DIR‐S/O/W‐E, and S/O/W‐E‐DIR, respectively. Fluorescence imaging of mice was performed at specific time intervals (2, 6, 12, and 24 h) using a spectrum in vivo imaging system (IVIS Lumina X RMS, PerkinElmer, USA) with an excitation wavelength of 760 nm and an emission wavelength of 790 nm. Then, the intestinal tract of mice was separated, and the intestinal distribution of S/O/W‐E was observed via the in vivo imaging system. The fluorescence intensity of mice and intestinal tissues was evaluated using Living Image Software. Ethical approval was granted by the Animal Ethics Committee of Ocean University of China.

### 2.7 In Vivo Bioavailability Evaluation

After 1 week of acclimatization, four‐week‐old male ICR mice (20 ± 2 g) were divided into three groups and gavaged with FUC suspension, Cur suspension, and FUC‐Cur‐S/O/W‐E (10 mg/kg FUC body weight, 25 mg/kg Cur body weight), respectively. Subsequently, all mice were allowed food and water ad libitum. Blood samples were obtained from the eyeballs of mice (six mice per group) into EDTA‐containing microtubes at 0.5, 1, 2, 4, 6, 8, and 12 h after oral gavage, and centrifuged (4°C, 5000 rpm, 10 min) to collect plasma (Wang et al. 2021). Ethical approval was granted by the Animal Ethics Committee of Ocean University of China.

To extract Cur, 100 μL of plasma with the addition of 250 μL ethyl acetate was vortexed for 60 s, and the supernatant was collected after centrifuging (4°C, 10,000 rpm, 5 min). In addition, FUC and its metabolites were extracted referring to the method of Li et al. (2021). In brief, 100 μL of plasma was mixed with 400 μL dichloromethane/methanol mixture (1:2, v/v) and vortexed for 60 s. Following this, 200 μL of n‐hexane was added and vortexed for 30 s. The mixture was then centrifuged (4°C, 1000 g, 5 min), and the resulting upper phase was collected. All the above extraction processes were repeated twice. The combined extracts were blow‐dried with nitrogen, redissolved in 100 μL methanol, and detected after filtration. Plasma concentration of Cur or FUC was quantified by an HPLC system according to method 2.4.

### 2.8 Statistical Analysis

Data were expressed as the mean value ± standard deviation. Statistical analysis was performed using one‐way analysis of variance (one‐way ANOVA), and *p* < 0.05 was considered statistically significant.

## 3 Results and Discussion

### 3.1 Characterization of FUC‐Cur‐S/O/W‐E in Simulated Gastrointestinal Conditions

**FIGURE 2:**
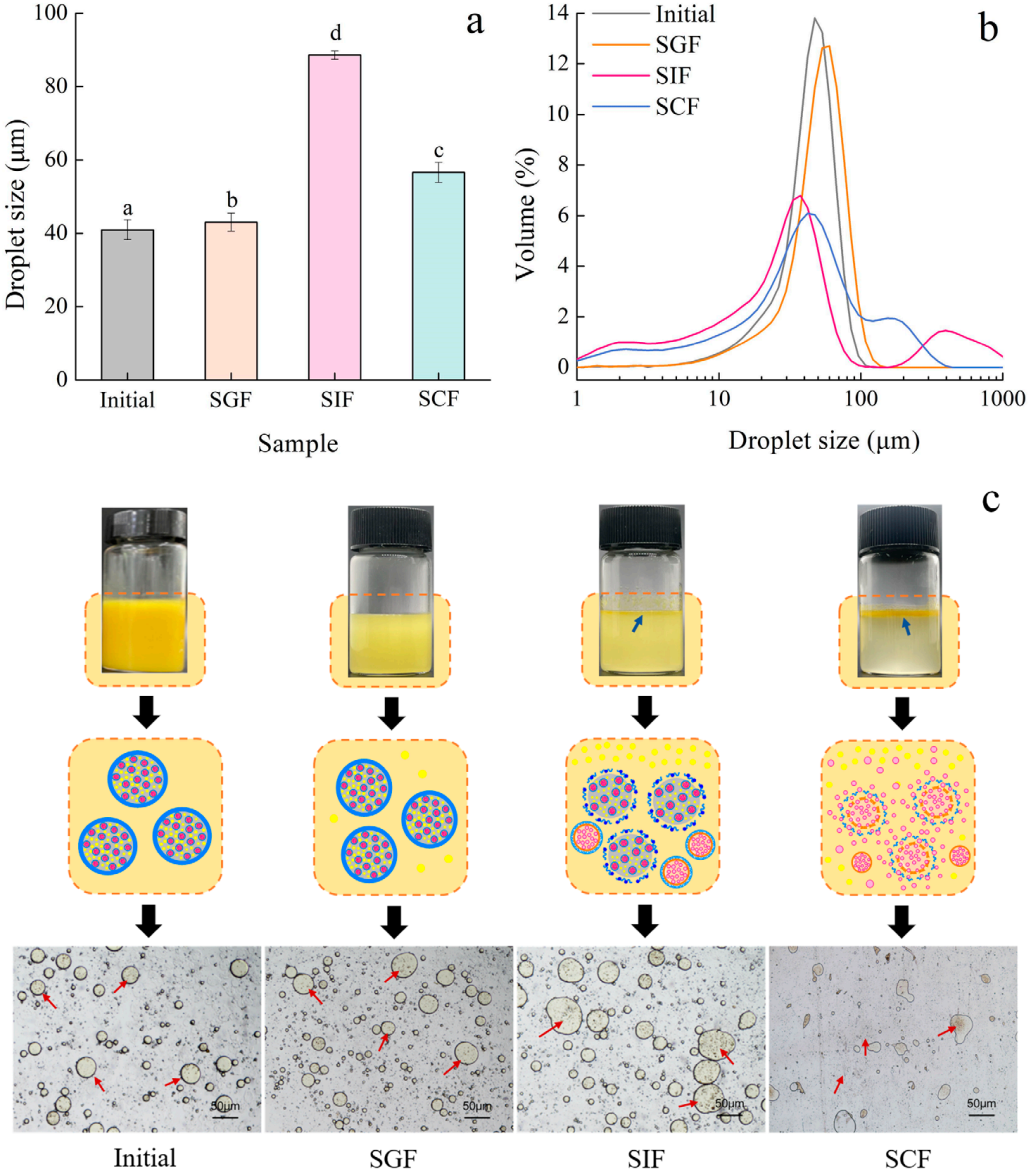
Droplet size (a), size distribution (b), and morphology (c) of FUC‐Cur‐S/O/W‐E in simulated gastrointestinal fluids.

The absorption of bioactives loaded in emulsions has been reported to depend on their changes in the GIT (McClements and Li 2010). Figure 2a,b shows the changes in droplet size and size distribution of FUC‐Cur‐S/O/W‐E in simulated gastrointestinal fluids. The droplet size of the initial untreated FUC‐Cur‐S/O/W‐E was 40.9 μm, and the size distribution showed a single symmetric peak. After digestion in SGF for 2 h, the droplet size of FUC‐Cur‐S/O/W‐E increased slightly, and the size distribution pattern remained unchanged. S/O/W‐E after digestion in SGF remained homogenous in appearance (Figure 2c), and there was no obvious oil layer. Combined with the micrographs, it was observed that the emulsion droplets were relatively regular and round, and the NPs could be clearly seen encapsulated inside the emulsion droplets, which indicated that S/O/W‐E could remain relatively stable in SGF. The dense interfacial membrane of the outer W phase protected S/O/W‐E from pepsin digestion and the harsh environment of SGF. CMS can be protonated under acidic conditions while maintaining a compact conformation, with further protection afforded by the spatial repulsion of PGA (Zhang et al. 2021). Moreover, the CMS/PGA complex as the W phase prevents pepsin adsorption. The high steric hindrance and surface activity of PGA create a competitive environment, resulting in limited enzyme binding to the droplet surface (Sharma et al. 2021; Yilmazer et al. 1991).

A significant increase in the droplet size of FUC‐Cur‐S/O/W‐E was found after 3 h of digestion in SIF. Some smaller‐sized droplets appeared along with the broadening of the size distribution (Figure 2b). Some aggregates appeared in S/O/W‐E with a transparent oil layer at the top, and the droplets could be observed to become irregular on the micrographs (Figure 2c), suggesting that the CMS/PGA complex as the outer W phase was eroded in SIF, and the S/O phase of S/O/W‐E was exposed. Released Cur and FUC NPs from the exposed S/O phase and free fatty acids from lipid digestion deposited on the emulsion droplet surface to form a large number of colloidal mixtures (e.g., mixed micelles, vesicles and insoluble complexes) (Hur et al. 2009) might lead to the emergence of aggregation, which explained the increased droplet size of S/O/W‐E in SIF. It should be noted that this may represent a plausible mechanism based on the observed droplet size changes. Direct analysis of the lipolysis process would be required in future work to confirm the extent of lipid digestion and its precise role in mediating these colloidal interactions.

The droplet size of S/O/W‐E tended to decrease after digestion in SCF (Figure 2a), when the emulsion structure was completely disrupted and the size distribution was uneven (Figure 2b). After digestion in SCF, the system presented two distinct layers, with a turbid chowder and suspension in the upper layer and a transparent lower layer. The dispersed granular material and deformed droplets in the system can also be seen in the micrograph of Figure 2c. It was hypothesized that the S phase (FUC NPs) exposed in SIF underwent degradation in SCF, leading to the reduction of large particles of aggregates associated with NPs, which further induced a change in the appearance and morphology of S/O/W‐E.

### 3.2 Release Profiles of FUC‐Cur‐S/O/W‐E in Simulated Gastrointestinal Conditions

**FIGURE 3:**
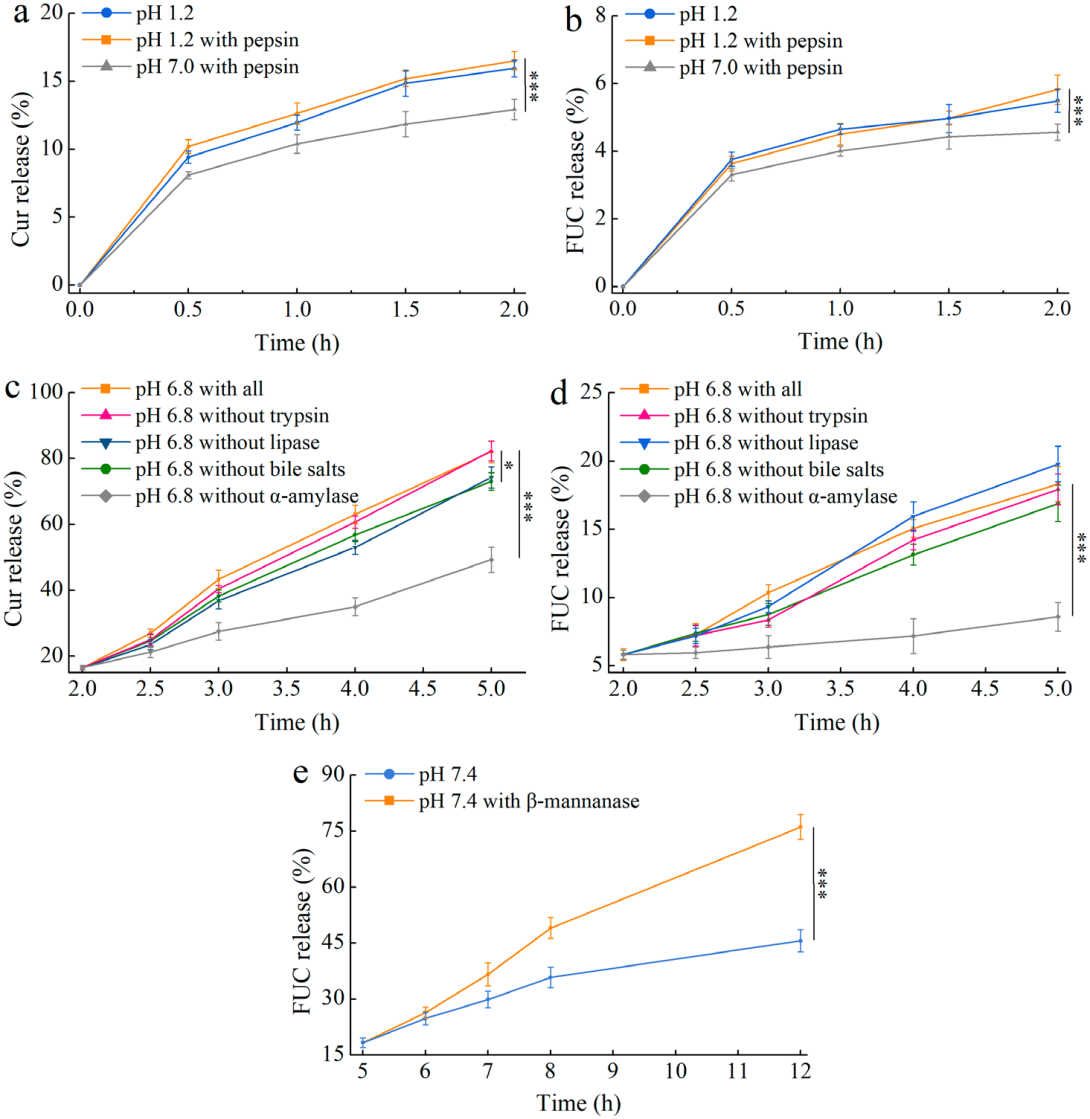
Effects of SGF (a, Cur; b, FUC), SIF (c, Cur; d, FUC) and SCF (e, FUC) on the release behavior of Cur and FUC in FUC‐Cur‐S/O/W‐E. *p < 0.05, **p < 0.01 and ***p < 0.001.

In vitro digestion models with different formulations of simulated gastrointestinal fluids were used to determine differences in release profiles of S/O/W‐E to further explore the GIT factors affecting the programmed sequential release behavior of S/O/W‐E. Effects of SGF on the release behavior of Cur and FUC in FUC‐Cur‐S/O/W‐E are depicted in Figure 3a,b. The acidic environment of SGF exerted a more pronounced effect on the release behavior of Cur in S/O/W‐E compared with pepsin (Figure 3a), which was related to the property of the outer W phase of S/O/W‐E. As a polysaccharide complex system, the CMS/PGA complex was not degraded by pepsin (Wang et al. 2022b). Compared to pH 7.0, S/O/W‐E showed a higher Cur release rate in SGF at pH 1.2, indicating that low pH affected the structure of S/O/W‐E stabilized by CMS/PGA complexes, especially on CMS. It has been reported that starch structure is susceptible to disruption in acidic environments (Warren et al. 2015). As can be seen from Figure 3b, the SGF composition showed a minimal influence on the release of FUC from FUC‐Cur‐S/O/W‐E. The FUC released in SGF after 2 h of digestion was all less than 6% owing to the fact that the FUC located in the innermost S phase of S/O/W‐E was protected by multiple interfacial layers.

Influence of trypsin, lipase, α‐amylase and bile salts in SIF on the release behavior of Cur and FUC in S/O/W‐E is depicted in Figure 3c,d. Obviously, α‐amylase significantly affected the release behavior of FUC‐Cur‐S/O/W‐E, with only 32.8% and 2.2% release of Cur and FUC after digestion in SIF without added α‐amylase. The α‐amylase‐free SIF reduced the hydrolysis of CMS/PGA complexes as the W phase, and the relatively intact structure of S/O/W‐E weakened the release of Cur from the O phase while decreasing the contact of FUC located in the S phase with simulated gastrointestinal fluids, which in turn led to a decline in FUC release. Compared to lipase and bile salts, trypsin exerted less influence on the release behavior of Cur in S/O/W‐E, as the outer W phase of S/O/W‐E belongs to a polysaccharide complex and is resistant to protease digestion. Digestion in SIF without bile salts or lipase reduced the amount of Cur released. Bile salts play a crucial role in emulsifying fats, enhancing lipase binding to the oil interface, and facilitating fat digestion. Prior studies revealed that bile salts would compete with pre‐adsorbed emulsifiers at the oil–water interface during digestion, allowing more lipase to participate in fat catabolism and thus promoting micelle formation (Guan et al. 2023). Mixed micelles present in digestive fluids could improve the bioaccessibility of bioactive components encapsulated in delivery systems (Xu et al. 2017). Further research characterizing the physicochemical properties (e.g., size and composition) of these mixed micelles would be valuable to fully elucidate their role in the absorption process. As a result, the S/O/W‐E structure in SIF was disrupted, and the exposed O phase was lipolyzed by lipase. At the same time, bile salts further accelerated its digestion (Li et al. 2012), contributing to a significant release of Cur, which explains that the absence of bile salts or lipase reduces the Cur release from S/O/W‐E. Compared to Cur, trypsin and bile salts had less influence on the release behavior of FUC in the S phase. Notably, FUC release rate tended to increase after digestion in SIF without added lipase, due to the hydrolysis of FUC by lipase (Wang et al. 2020). The lipase present in the GIT can hydrolyze the free FUC released from FUC‐Cur‐S/O/W‐E into its metabolite form, fucoxanthinol (FxOH), resulting in small results for FUC release measured in the lipase‐containing SIF formulation.

Figure 3e revealed that β‐mannanase in SCF remarkably affected FUC release in FUC‐Cur‐S/O/W‐E. Digestion in SCF without β‐mannanase greatly reduced the amount of FUC released, and it was hypothesized that the FUC release in SCF was governed by the erosion of β‐mannanase (Wang et al. 2022a). After digestion by SGF and SIF, the FUC NPs entrapped in S/O/W‐E as the S phase were fully exposed, and the coating material of FUC NPs, CMK, was degraded by the β‐mannanase in SCF, causing massive FUC release.

### 3.3 Release Kinetics and Mechanism of FUC‐Cur‐S/O/W‐E

**FIGURE 4:**
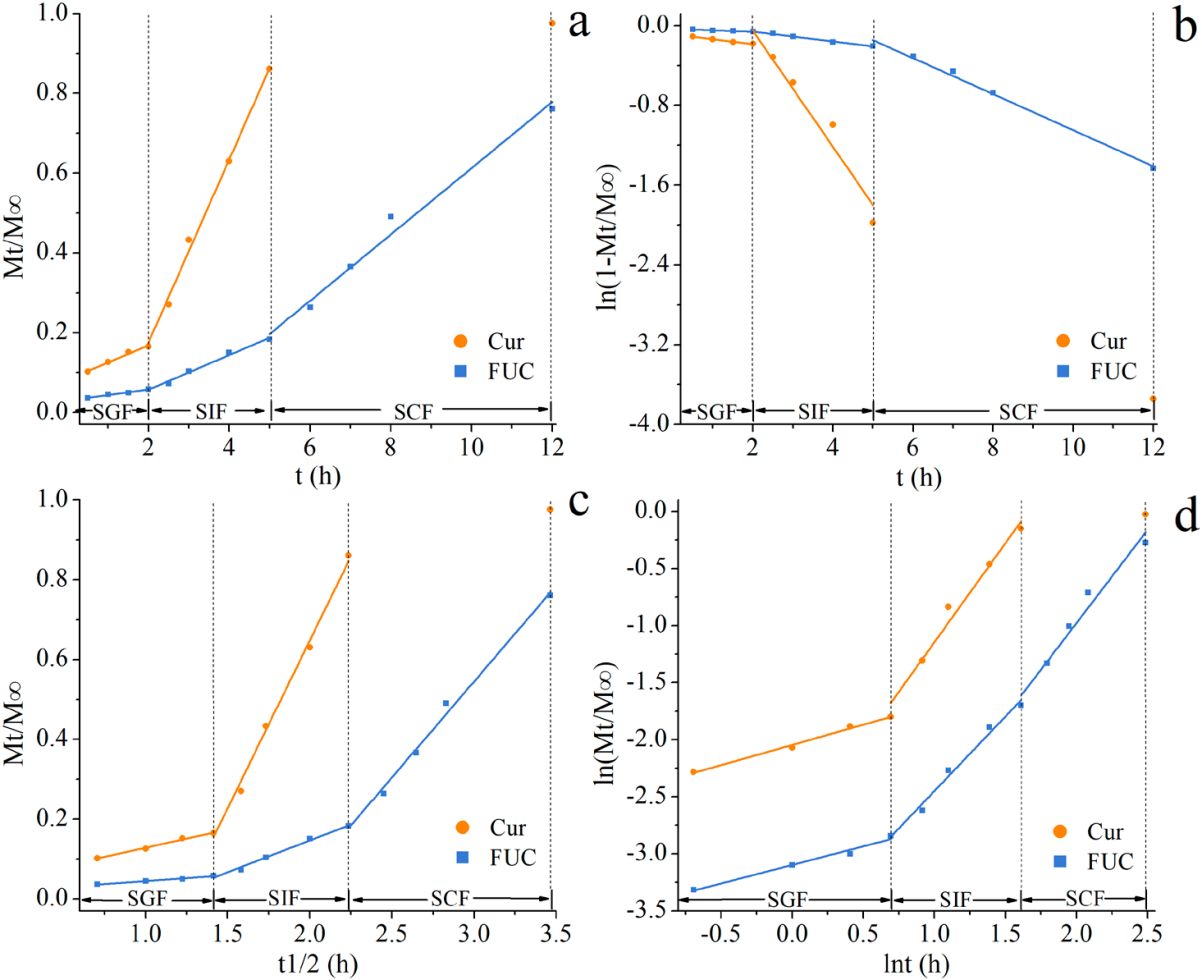
Kinetics fitting curves of Cur and FUC releases from FUC‐Cur‐S/O/W‐E in simulated gastrointestinal fluids (a, Zero‐order model; b, First‐order model; c, Higuchi model; d, Korsmeyer–Peppas model).

The release kinetics of Cur and FUC from S/O/W‐E in SGF, SIF, and SCF were investigated by Zero‐order, First‐order, Higuchi, and Korsmeyer‐Peppas release kinetic models. The optimal kinetic model was determined comprehensively based on the adjusted *R* ^2^ value and the Akaike Information Criterion (AIC). Figure 4 displays the release curves fitted by different kinetic models, and the release kinetic parameters of Cur and FUC encapsulated in S/O/W‐E in simulated gastrointestinal fluids are listed in Table 1.

**TABLE 1:** Kinetic parameters of Cur and FUC releases from FUC‐Cur‐S/O/W‐E in simulated gastrointestinal fluids.

	Model		SGF	SIF	SCF
Cur	Zero‐order model	*k*	0.04298	0.22953	—
		Adjusted *R* ^2^	0.97478	0.99468	
		AIC	−44.1036	−30.3663	—
	First‐order model	Adjusted *R* ^2^	0.97749	0.93293	
		AIC	−43.4087	−13.4310	—
	Higuchi model	Adjusted *R* ^2^	0.99083	0.99277	
		AIC	−48.1494	−30.1634	—
	Korsmeyer‐Peppas model	*n*	0.35438	1.73776	—
		Adjusted *R* ^2^	0.9899	0.97757	
		AIC	−29.4967	−31.5842	—
FUC	Zero‐order model	*k*	0.01409	0.04343	0.08312
		Adjusted *R* ^2^	0.98139	0.98669	0.98205
		AIC	−54.2597	−40.0348	−27.4128
	First‐order model	Adjusted *R* ^2^	0.98146	0.98912	0.99053
		AIC	−53.8848	−39.9452	−26.2236
	Higuchi model	Adjusted *R* ^2^	0.97348	0.98869	0.99205
		AIC	−52.8421	−41.8397	−30.4701
	Korsmeyer‐Peppas model	*n*	0.32539	1.30694	1.63383
		Adjusted *R* ^2^	0.97236	0.9836	0.96139
		AIC	−49.1055	−41.8841	−30.6181

All four kinetic models were fitted with high *R* ^2^ values, showing that all of the above models were capable of describing bioactive release from S/O/W‐E over time during the simulated digestion. The release rate of S/O/W‐E is determined by the Zero‐order model with *R* ^2^ values above 0.97. The *k* value represents the release rate of the encapsulated bioactive component, which is positively correlated with the release rate (Fan et al. 2020). The *k* value of Cur in S/O/W‐E was higher in SIF than in SGF, showing a faster release rate of Cur in SIF than in SGF, indicating that the outer W phase was degraded in SIF, and the S/O/W‐E structure was disrupted. Compared to SGF and SIF, the *k* value of FUC was significantly larger in SCF, demonstrating that FUC was released faster in SCF, which was related to the degradation of S phase (FUC NPs) in this environment.

Elucidating the release mechanism of the constructed delivery systems is essential for their application. The release mechanism of the delivery system describes the release mode of the encapsulated bioactive components, which typically includes diffusion, dissolution, swelling, and erosion (Wen et al. 2017), depending on the properties of the carrier material and external environment. The Korsmeyer‐Peppas model utilizes the *n* value to conclude the release mechanism of bioactive components. For the Korsmeyer‐Peppas model, *n* < 0.43 denotes a Fickian release (case I transport), showing a diffusion release mechanism. Further, *n* > 0.85 indicates a Fickian release (case II transport), showing an erosion release mechanism. In addition, 0.43 < *n* < 0.85 suggests a non‐Fickian release, describing the concurrent release mechanism of diffusion and erosion (Ma et al. 2021). Figure 4d shows the release data of Cur and FUC in simulated gastrointestinal fluids from S/O/W‐E fitted by the Korsmeyer‐Peppas model. The Cur and FUC released during simulated digestion fitted well with the Korsmeyer‐Peppas model, with *R* ^2^ values greater than 0.96. According to Table 1, the *n* value of Cur released in SGF was lower than 0.43, following a Fickian release (case I transport) dominated by diffusion. With *n* value of Cur exceeding 0.85 in SIF, Cur release from S/O/W‐E displayed a Fickian release (case II transport) governed by erosion, owing to the loose structure of S/O/W‐E under SIF conditions. Furthermore, the *n* value of FUC in SGF was found to be lower than 0.43, indicating the presence of a Fickian diffusion mechanism and that the FUC release of S/O/W‐E in SGF was mainly a diffusion process. In SIF and SCF, the *n* values of the FUC release profiles were more than 0.85, showing a case II transport type, which suggested that FUC in S/O/W‐E was controlled mainly by erosive release in both SIF and SCF.

**FIGURE 5:**
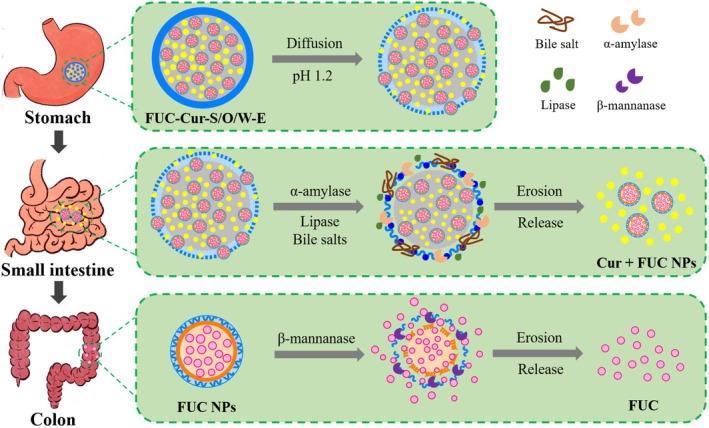
Schematic representation of the release mechanism of Cur and FUC in FUC‐Cur‐S/O/W‐E.

Taking the above results together, the mechanism of Cur and FUC release from S/O/W‐E is summarized in Figure 5. S/O/W‐E is relatively stable in the gastric conditions, and the co‐encapsulated Cur and FUC are released in the stomach in small amounts, relying on diffusion mechanisms. The release of Cur is unaffected by pepsin but accelerated by the acidic environment, while the release of FUC is less affected by the gastric environment. Upon transit to the small intestine, the outer W phase is degraded, and the S/O/W‐E structure is disrupted. A large amount of Cur is released from the exposed S/O phase, with an erosive release mechanism predominating. Hydrolysis by α‐amylase, digestion by lipase and bile salts in the small intestine accelerate Cur release from S/O/W‐E. In contrast, the FUC release in the small intestine is relatively small. FUC located in the S phase of S/O/W‐E is massively released upon arrival in the colon and is dominated by erosion. The β‐mannanase in the colon is the main factor contributing to the massive release of FUC.

### 3.4 In Vivo Fluorescence Imaging of Res‐Vd‐S/O/W‐E in Mice

**FIGURE 6:**
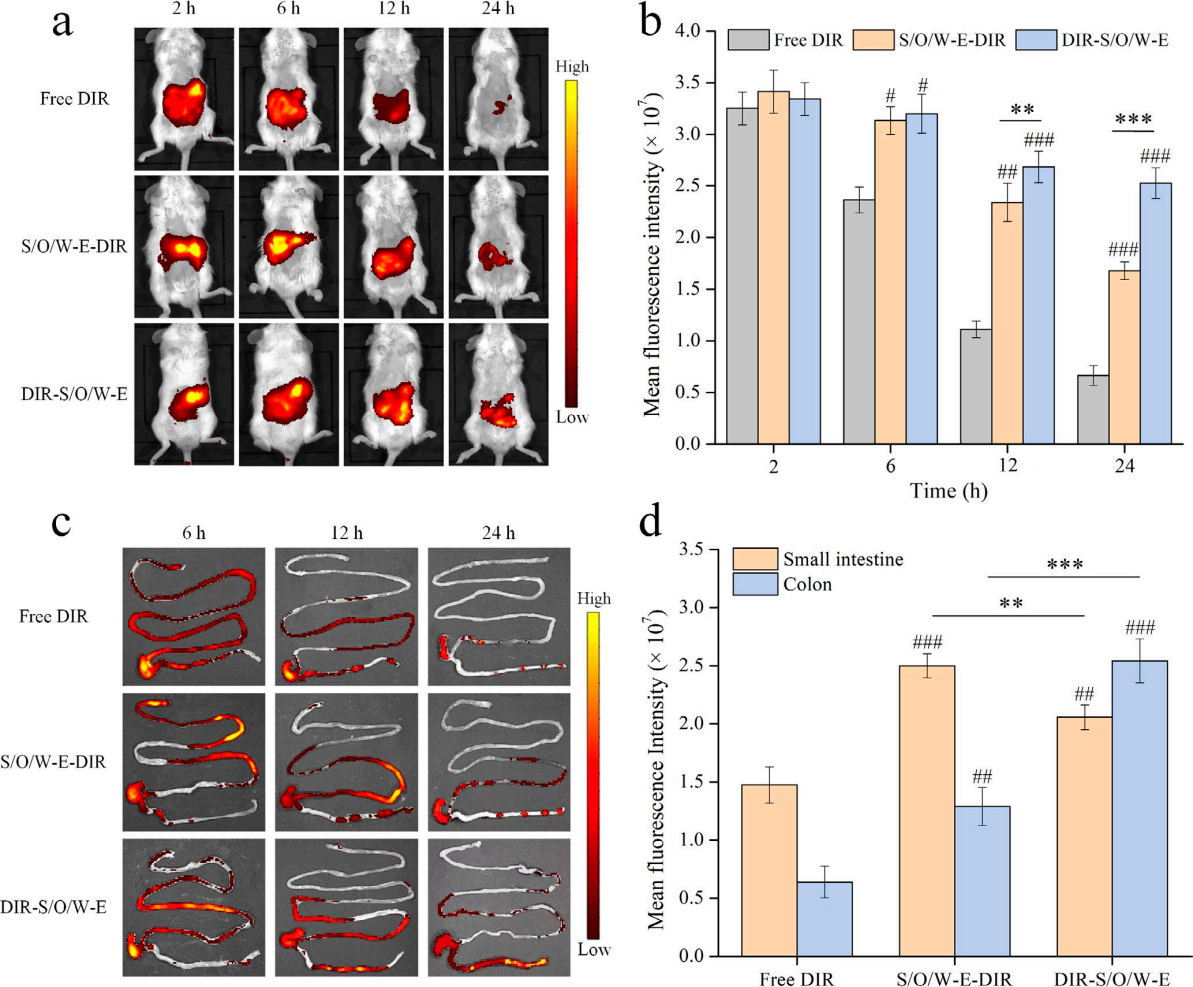
Fluorescence images of mice (a) and fluorescence intensity statistics (b) after oral administration of S/O/W‐E at 2, 6, 12, and 24 h. Fluorescence images of the small intestine and colon extracted from mice after oral administration of S/O/W‐E at 6, 12, and 24 h (c). Fluorescence intensity statistics of the small intestine after 6 h and colon after 24 h of oral administration of S/O/W‐E (d). ^#^ *p* < 0.05, ^##^ *p* < 0.01 and ^###^ *p* < 0.001 vs Free DIR. *p < 0.05, **p < 0.01 and ***p < 0.001.

DIR was selected as a fluorescence tracer to replace bioactive components (Res and Vd), and an in vivo imaging system was employed to visually observe the distribution of orally administered S/O/W‐E within mice. Figure 6a,b showed that mice in the free DIR group exhibited a broader fluorescence distribution after 2 h of oral administration, and the whole‐body fluorescence intensity of mice decreased remarkably with the prolongation of oral administration time. Compared with the S/O/W‐E group, the free DIR group fluoresced extremely weakly after 12 h and almost completely disappeared after 24 h, with only minimal fluorescence intensity detected. In contrast, the fluorescence intensity of the S/O/W‐E‐DIR group and the DIR‐S/O/W‐E group showed significant differences from that of the free DIR group at 6, 12, and 24 h, indicating that S/O/W‐E slowed down the rapid metabolism of DIR in vivo. The fluorescence intensity of mice in the S/O/W‐E‐DIR group was dramatically higher than that in the DIR‐S/O/W‐E group after oral administration for 12 and 24 h, which was related to the different locations of DIR encapsulated in S/O/W‐E, showing that the release of DIR encapsulated in the S/O/W‐E‐DIR group was earlier than that of the DIR‐S/O/W‐E group in the GIT.

Further, differences in intestinal distribution of bioactive components encapsulated in different locations of S/O/W‐E were explored by isolating mouse intestines (Figure 6c,d). Consistent with the results of the whole‐body fluorescence experiments conducted on mice, the intestinal fluorescence intensity of mice in the S/O/W‐E group was substantially higher than that of the free DIR group after 12 and 24 h of oral administration (Figure 6c), demonstrating that encapsulating DIR into S/O/W‐E prolonged its retention time in the intestinal tract. As illustrated in Figure 6d, the small intestine site of mice in the S/O/W‐E‐DIR group showed the highest fluorescence intensity after 6 h of administration. In comparison, the colon site of mice in the DIR‐S/O/W‐E group displayed the highest fluorescence intensity after 24 h. It confirmed that the different bioactive components encapsulated in the O phase and the S phase of S/O/W‐E could be enriched in the small intestinal and colon tissues, respectively. In summary, in vivo fluorescence imaging experiments in mice visually corroborated the results of in vitro simulated release, demonstrating that the constructed S/O/W‐E could sequentially deliver the two different bioactive components encapsulated in the oil phase and solid phase to the small intestine and colon, respectively.

### 3.5 Pharmacokinetics Analysis of FUC‐Cur‐S/O/W‐E in Mice

**FIGURE 7:**
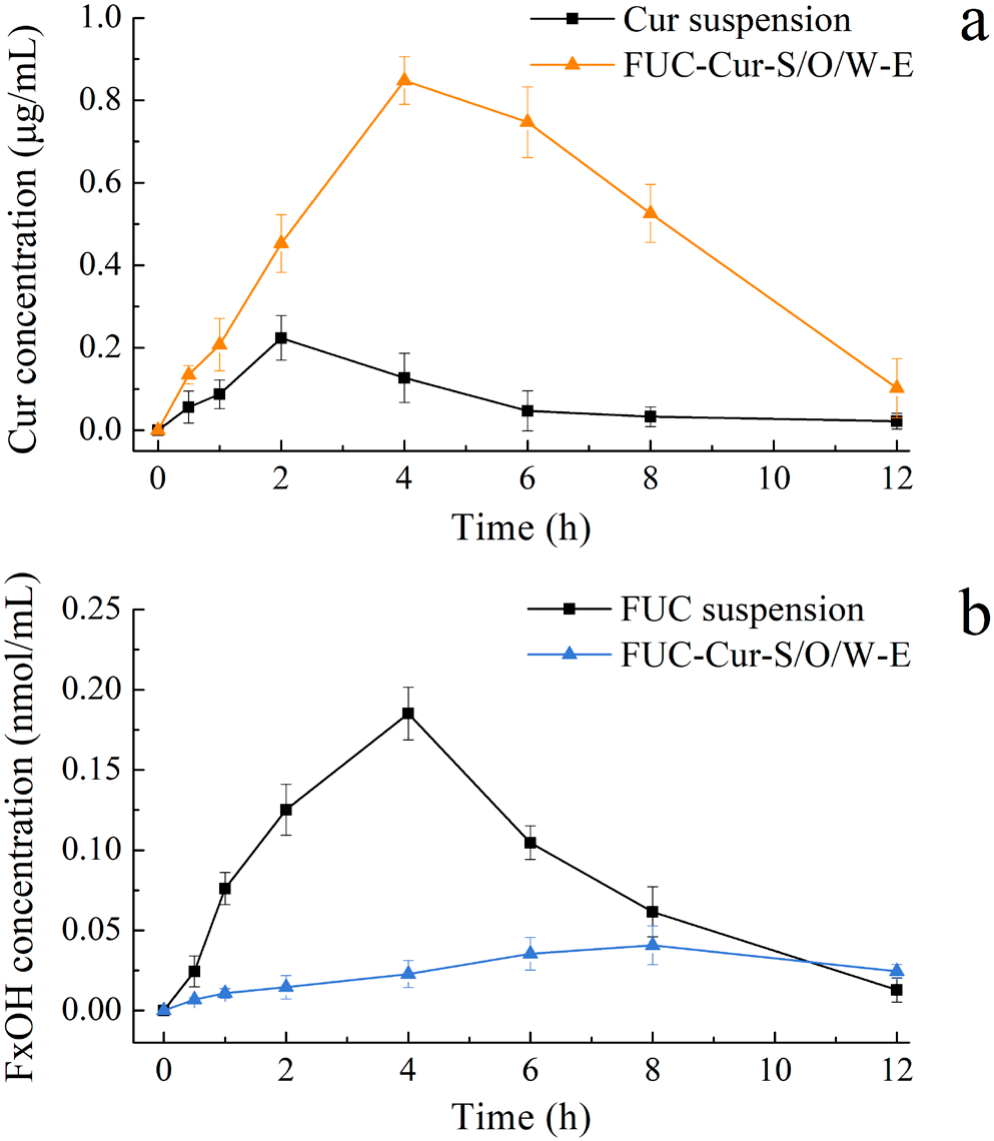
Plasma level‐time profiles of Cur (a) and FxOH (b) after oral administration of FUC‐Cur‐S/O/W‐E.

The pharmacokinetics of Cur and FUC were studied in mice after oral gavage of FUC‐Cur‐S/O/W‐E. Changes in plasma concentration of Cur and FxOH over time and their pharmacokinetic parameters are illustrated in Figure 7 and Table 2, respectively. The maximum plasma concentration (*C*_max_) of Cur in the Cur suspension group was 0.224 μg/mL, which appeared at 2 h after gavage, and the area under the curve (AUC_0‐12_) was 0.916 h·μg/mL. The time to reach *C*_max_ (*T*_max_) of free Cur corresponds to that reported by Chavez‐Zamudio et al. (2017). For the S/O/W‐E group, the *C*_max_ was 0.848 μg/mL and appeared at 4 h post‐gavage, and the AUC_0‐12_ was 5.877 h·μg/mL. The Cur bioavailability in the S/O/W‐E group was 5.4‐fold higher than that in the Cur suspension group, implying that Cur delivered by S/O/W‐E was more efficiently absorbed than that in the free state. Lipid‐based delivery systems (e.g., microemulsions, nanoemulsions and lipid NPs) have been reported to promote micellarized transport and intestinal absorption of lipophilic bioactive components (McClements 2018). The study of Roodenburg et al. (2000) demonstrated that a high‐fat diet (207% increase) improved plasma lutein levels than a low‐fat diet (88% increase) with intake of lutein esters. Loading vitamin D into oil‐in‐water nanoemulsions could increase its bioavailability by 80% (Ozturk et al. 2015). Encapsulation of β‐carotene in variously sized emulsions led to a 34%–59% increase in bioavailability (Salvia‐Trujillo et al. 2013). In fact, the interaction of dietary lipids with bile salts and others in the gut during gastrointestinal digestion can form colloidal structures to dissolve lipophilic bioactive components (e.g., Cur) and transport them to epithelial cells to facilitate their absorption (Liu et al. 2019). The dense structure of S/O/W‐E constructed with CMS/PGA complex as the W phase could inhibit droplet aggregation and enhance the contact of lipase and bile salts with the O phase in the intestine, thus facilitating the transfer of the encapsulated bioactive components from the O phase to the micelles, and ultimately improving their bioaccessibility (Zhang et al. 2023).

**TABLE 2:** Pharmacokinetic parameters of Cur and FxOH after oral administration of FUC‐Cur‐S/O/W‐E.

Sample		*T*_max_	*C*_max_	AUC_0‐12_	MRT_0‐12_
Cur	Cur suspension	2	0.224	0.916	4.002
	FUC‐Cur‐S/O/W‐E	4	0.848	5.877	5.510
FxOH	FUC suspension	4	0.185	1.048	4.739
	FUC‐Cur‐S/O/W‐E	8	0.041	0.321	6.932

*Note:* The units of *T*_max_ and MRT_0‐12_ are h. The units of *C*_max_ for Cur and FxOH are μg/mL and nmol/mL, respectively. The units of AUC_0‐12_ for Cur and FxOH are h·μg/mL and h·nmol/mL, respectively.

As free FUC is readily hydrolyzed in the GIT or converted to FxOH by lipase, cholesterol esterase and carboxyl esterase during digestion (Liu et al. 2019), the pharmacokinetic study of S/O/W‐E encapsulated FUC is expressed in the form of FxOH. The *T*_max_ of free FUC was 4 h, which is consistent with the finding of Koo et al. (2016). Encapsulation of FUC into S/O/W‐E significantly prolonged its *T*_max_, which was extended to 8 h in the FUC‐Cur‐S/O/W‐E group. A short *T*_max_ indicates rapid absorption and elimination of the bioactive components, whereas a long *T*_max_ indicates slower absorption of the bioactive component and a longer duration of action. Similar to the colon‐targeted delivery system (Rai et al. 2016), the degradation of the delivery system by the enzymes and microflora present in the colon is a slow process, leading to a prolonged time of release and action of the encapsulated bioactive components, which also confirms that S/O/W‐E could target bioactive components encapsulated in the S phase to the colon. The *C*_max_ of FUC suspension reached about 0.185 nmol/mL after gavage for 4 h, while that of the S/O/W‐E encapsulated FUC was only 0.041 nmol/mL. Obviously, compared with most of the free FUC being absorbed into the blood in the upper GIT, only a small amount of FUC in FUC‐Cur‐S/O/W‐E was released into the small intestine, and its hydrolytic transformation in the GIT and its entry into the blood circulation were less. The above results indicated that S/O/W‐E could increase the bioavailability of bioactive components in the O phase, reduce the release of bioactive components located in the innermost S phase in the upper GIT, and realize their delivery in the colon.

## 4 Conclusions

In summary, FUC‐Cur‐S/O/W‐E co‐encapsulated Cur and FUC were successfully constructed by using FUC NPs as the S phase, Cur‐containing coconut oil as the O phase, and CMS/PGA complexes as the W phase through the structural design principle. Changes of S/O/W‐E during in vitro digestion, the release kinetics and mechanisms of encapsulated Cur and FUC in the simulated gastrointestinal environment, and their bioavailability in vivo were evaluated. It was shown that S/O/W‐E was relatively stable in the gastric environment. The release of co‐encapsulated Cur and FUC in SGF depended on the diffusion mechanism, and the acidic environment accelerated Cur release from the O phase. Cur was released in large quantities in SIF and predominantly by erosion, which was facilitated by α‐amylase, lipase, and bile salts. Due to the presence of β‐mannanase, a large amount of FUC was released from the S phase in SCF and dominated by the erosive mechanism. In addition, in vivo fluorescence imaging and pharmacokinetic analyzes revealed that S/O/W‐E was capable of delivering bioactive components encapsulated in the O phase to the small intestine, and increasing their bioavailability in vivo. Furthermore, S/O/W‐E inhibited the release of bioactive components encapsulated in the S phase in the upper GIT while delivering a substantial amount to the colon. The current research further provides a theoretical foundation for expanding the application of S/O/W‐E in the co‐delivery and programmed sequential release of different bioactive components.

## Author Contributions

**Changhu Xue:** conceptualization, resources, supervision, funding acquisition, project administration. **Luhui Wang:** conceptualization, methodology, data curation, investigation, formal analysis, funding acquisition, project administration, writing – original draft, writing – review and editing, visualization. **Mingqing Wang:** methodology, resources, writing – review and editing. **Ling Lv:** methodology, investigation, writing – review and editing.

## Funding

This work was supported by the Taishan Scholars Program of Shandong Province (Grant tsqnz20250761), Innovation Project of Agricultural Science and Technology of Shandong Academy of Agricultural Sciences (Grant CXGC2025F21‐1‐19), Key R&D Program of Shandong Province (Grant 2023TZXD074), and Science and Technology Services by Science and Technology Commissioners (Grant 2022DXAL0102).

## Ethics Statement

This study was approved by the Institutional Review Board of Ocean University of China.

## Conflicts of Interest

The authors declare no conflicts of interest.

## Data Availability

The data that support the findings of this study are available from the corresponding author upon reasonable request.
