# Correction to “Programmed Sequential Release Behavior and Bioavailability of Curcumin and Fucoxanthin Co‐Encapsulated in Solid‐in‐Oil‐in‐Water Multilayer Emulsions”

**DOI:** 10.1002/fsn3.72175

**Published:** 2026-07-29

Wang L , Wang M , Lv L , et al. Programmed Sequential Release Behavior and Bioavailability of Curcumin and Fucoxanthin Co‐Encapsulated in Solid‐in‐Oil‐in‐Water Multilayer Emulsions. Food Science and Nutrition, 2026, 14(1):e71463. 10.1002/fsn3.71463 41567167 PMC12816879.

In the author affiliations section, the first affiliation was updated to “^1^Shandong Academy of Agricultural Sciences, Jinan, China”.

The online version of this article has been corrected accordingly.

We apologize for this error.

